# Editorial: Insights in Rheumatology: 2021

**DOI:** 10.3389/fmed.2022.971777

**Published:** 2022-07-22

**Authors:** João E. Fonseca, Ana Margarida Duarte-Monteiro

**Affiliations:** ^1^Rheumatology Department, Centro Hospitalar Universitário Lisboa Norte, Lisbon Academic Medical Centre, Lisbon, Portugal; ^2^Instituto de Medicina Molecular João Lobo Antunes, Faculdade de Medicina, Universidade de Lisboa, Lisbon, Portugal

**Keywords:** IL-33, B cells, synovial fibroblasts, regenerative medicine, pre RA

In this Research Topic, entitled *Insights in Rheumatology: 2021*, a broad range of subjects were highlighted, from fibromyalgia to gout, also encompassing novelties on the role of Interleukin-33 (IL-33) and new treatment paradigms such as regenerative medicine, giving a glimpse of the heterogeneity of rheumatic diseases.

In a very interesting and stimulating manuscript, Maugars et al. highlighted the concept of hypersensitization as a way of integrating a complex symptom constellation that goes beyond generalized pain in the context of fibromyalgia. The authors also discuss the role of rheumatologists in the approach to this condition, emphasizing the relevance of the differential diagnosis. Other concepts such as coping, resilience, catastrophizing, and the possible relationship with post-traumatic syndromes underline the psychologic dimension of the fibromyalgia patient. Furthermore, neuro-imaging findings have allowed a better understanding of the pathology of fibromyalgia as a disorder of the cortical integration of chronic pain, with amplification of sensory nociception signals and a decrease in pain perception threshold, combined with a persistence of the stimulus that contributes to chronicity (Maugars et al.). Gout is another very frequent challenge facing rheumatologists and it is arguably one of the “oldest” rheumatic diseases. However, we still face patients where available treatment options fail to completely control the disease. Thus, it is clearly of interest to understand the clinical experience of a gout Chinese cohort treated with benzbromarone (a nonpurine xanthine oxidase inhibitor), a first-line urate-lowering drug in Asia (Xue et al.).

Rheumatology is still seeking the full understanding of the cytokine networks that are behind chronic immune mediated inflammatory diseases (IMID). IL-33 is a very interesting cytokine in this context. It can be expressed in inflammatory cells and play an immunomodulatory role, activating different cells and inducing cytokine production and thus inflammation through a pathway initiated by the IL-33/serum stimulation-2 (ST2) axis (Dong et al.). This axis has been shown to play a role in the pathophysiology of several rheumatic diseases, including gout, systemic lupus erythematosus (SLE), rheumatoid arthritis (RA) and systemic sclerosis (SSc) (Dong et al. and Versace et al.). Of interest, in the context of SSc, levels of IL-33 correlate with skin fibrosis, and this cytokine seems to induce both cutaneous and pulmonary fibrosis *via* increased IL-13. Versace et al. have found that IL-13 and IL-33 levels were increased in SSc patients and seem to associate with measures of pulmonary disfunction. These observations further reinforce the role of IL-33 in rheumatic diseases and open the possibility of exploring it as a therapeutic target or as a biomarker.

The autoimmune range of the rheumatic diseases' physiopathology spectrum is closely linked to the biology of B cells. From a pragmatic view the effectiveness of biologic therapies that target B cells in these diseases proves the direct or indirect role of these cells in the diseases mechanisms of conditions such as SLE and RA (1). The use of Belimumab in SLE treatment and active lupus nephritis is an example. Parodis et al. have demonstrated that, conflicting with previous findings (2–4), belimumab can induce rapid and sustained decreases in some plasma cell subsets, which does not seem to relate to response to treatment. On the other hand, the expanding-returning pattern observed in memory B cells induced by belimumab seems to be evident in clinical responders. Additionally, authors have demonstrated that clinical response to belimumab was associated with preceding reductions of anti-dsDNA and increases in C3 and C4 levels. The role of B cells is still unclear in the context of juvenile idiopathic arthritis (JIA). However, in subsets of patients, the B cell depleting therapy rituximab can be effective (Moura and Fonseca). Yet a better understanding of the possible common physiopathology mechanisms between RA and polyarticular and extended oligoarticular is needed to further dissect the heterogeneity of JIA.

Synovial fibroblasts are classic players in RA and their role in perpetuating inflammation and leading ultimately to damage is well-known. Thus, targeting these cells, could be and additional approach to achieve and maintain remission in RA (5). Interestingly, these treatment strategy could be particularly beneficial for patients with pauci-immune synovial pathotypes with a poor response to standard therapy (Chu). Current treatment options are not able to specifically interfere with synovial fibroblasts. However, indirectly they can influence the behavior of these cells, as is the example of peficitinib (a JAK inhibitor) that seems to suppress synovial fibroblast migration *in vitro* and may induce fibroblast apoptosis (Chu). Another persistent topic of discussion in RA is the concept of flare. A role of preinflammatory mesenchymal (PRIME) cells has been suggested, as they could actively migrate into the joint and stimulate local inflammation, with a potential contribution of long-term persistence of synovial resident memory T cells in the joint to their homing (Bozzalla-Cassione).

While the role of disease activity indexes is crucial for defining a flare, “patient reported outcomes” have become increasingly relevant, creating the concepts of patient-based and physician-reported flare. On the other extreme of natural history of RA are the subtle initial symptoms that may signalize the very early stage of RA, or even a stage that corresponds to an immune disturbance (for example the presence of anti-citrullinated protein antibodies) that represents a risk for RA, still without an inevitable physiopathology and clinical fate. Despite well-defined classification criteria for RA that allow identification of patients even at early stages of the disease, and thus treat them and prevent disease progression and damage, no definite definitions and clinical approach to patients at-risk for RA are established. Risk is represented by an interaction between environmental, genetic, and immune factors, associated or not with symptoms such as unspecific arthralgias (6). Identification of these patients through a European Registry of at-risk people can prove invaluable to research aimed at preventing progression (Studenic et al.).

While rheumatic diseases have effective treatments available, and the concept of prevention of disease in at-risk individuals seem to be becoming relevant, irreversible damage can be and is often a reality. Yoshimi and Nakajima recall the potential role of regenerative medicine as a paradigm shift in treating rheumatic patients with poor prognosis. Recent research has focused on autologous Hematopoietic Stem Cell Transplantation, and EULAR recommends it for skin and lung disease in rapidly progressive SSc at risk of organ failure (Yoshimi and Nakajima). Mesenchymal stem cell transplantation has been tried in SSc patients with improvement in skin sclerosis and ulcers, and in SLE patients refractory to standard therapy and lupus nephritis (Yoshimi and Nakajima). However, there is not sufficient evidence to formally recommend it in the clinical setting. More research is also for sure needed in the field of induced pluripotent stem cells and vascular endothelial progenitor cells.

Insights in Rheumatology 2021 bring us the richness of the progress in this field but also the unknown shadows that constitute the research agenda for the future.

## Author contributions

All authors listed have made a substantial, direct, and intellectual contribution to the work and approved it for publication.

## Conflict of interest

The authors declare that the research was conducted in the absence of any commercial or financial relationships that could be construed as a potential conflict of interest.

## Publisher's note

All claims expressed in this article are solely those of the authors and do not necessarily represent those of their affiliated organizations, or those of the publisher, the editors and the reviewers. Any product that may be evaluated in this article, or claim that may be made by its manufacturer, is not guaranteed or endorsed by the publisher.
